# Mechanism of Action of Icariin in Bone Marrow Mesenchymal Stem Cells

**DOI:** 10.1155/2019/5747298

**Published:** 2019-04-04

**Authors:** Aofei Yang, Chaochao Yu, Qilin Lu, Hao Li, Zhanghua Li, Chengjian He

**Affiliations:** ^1^Hubei Provincial Hospital of Traditional Chinese Medicine, Wuhan, 430061 Hubei, China; ^2^Hubei Province Academy of Traditional Chinese Medicine, Wuhan, 430070 Hubei, China; ^3^Hubei University of Chinese Medicine, Wuhan, 430061 Hubei, China; ^4^Hubei 672 Orthopaedics Hospital of Integrated Chinese & Western Medicine, Wuhan, 430079 Hubei, China; ^5^Tongren Hospital of Wuhan University, Wuhan, 430060 Hubei, China

## Abstract

Osteoporosis, femoral head necrosis, and congenital bone defects are orthopedic disorders characterized by reduced bone generation and insufficient bone mass. Bone regenerative therapy primarily relies on the bone marrow mesenchymal stem cells (BMSCs) and their ability to differentiate osteogenically. Icariin (ICA) is the active ingredient of *Herba epimedii*, a common herb used in traditional Chinese medicine (TCM) formulations, and can effectively enhance BMSC proliferation and osteogenesis. However, the underlying mechanism of ICA action in BMSCs is not completely clear. In this review, we provide an overview of the studies on the role and mechanism of action of ICA in BMSCs, to provide greater insights into its potential clinical use in bone regeneration.

## 1. Introduction

Conventional treatments for orthopedic disorders like osteoporosis, femoral head necrosis, bone defects, and nonunion disorders [1–7] have poor clinical efficacy due to their inability to ameliorate the loss in bone mass. Therefore, the current focus of treating bone disorders is tissue regeneration using bone marrow mesenchymal stem cells (BMSCs) [7, 8]. A number of studies have investigated the effects of various drugs, mechanical stress, physical stimuli, and scaffolds on BMSCs [9–11], in order to clinically translate its regenerative capacity [12]. Traditional Chinese medicine (TCM) has also garnered considerable interest in recent years due to its minimal toxicity [13]. According to the principles of TCM, bone function is closely associated with the balance of kidney yin and yang. Herbs such as *Herba epimedii*, *Fructus psoralea*, *Drynaria fortunei*, and *Radix dipsaci* are known to invigorate the kidney and restore the balance and are therefore frequently used to treat bone disease. Currently, *Herba epimedii* is the most deeply studied among those herbs in the bone regeneration field [14]. Icariin (ICA) is the main active ingredient of *Herba epimedii*, which has been used in TCM formulations to strengthen the muscles and bones [15]. Although *Herba epimedii* is still used to treat orthopedic disorders, its mechanism of action remains unclear. Several studies have analyzed the effects of ICA in BMSCs and other cells and provided new insights into its therapeutic role in orthopedic disorders. In this review, we have summarized the recent findings on the role and mechanism of action of ICA in BMSCs.

## 2. Basic Properties of ICA

**Figure 1:**
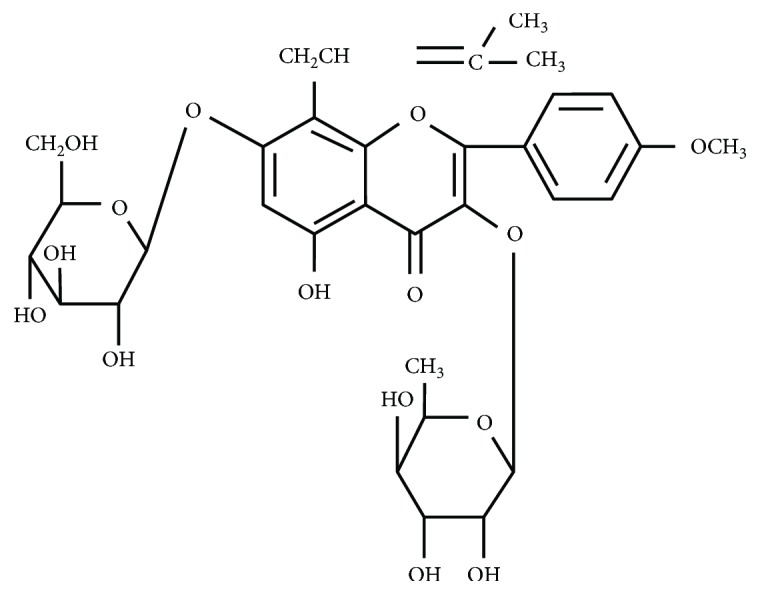
Chemical structure of ICA.

*Herba epimedii* (Yin Yang Huo in Chinese) is the dried leaf of *Epimedium brevicornum* Maxim as recorded in the Chinese Pharmacopoeia [16–18], as well as the 400-year-old Chinese medical classic *Shennong Ben Cao Jing*. It nourishes the kidney and significantly reinforces yang [18]. More than 20 flavonoids have been identified by chemical reaction and spectral analysis and isolated from *Herba epimedii* by systematical separation technology [17]. ICA (C_33_H_40_O_15_, molecular weight: 676.66, Figure 1) [17] is one of the primary active constituents which is also considered as the chemical marker for quality control components of *Herba epimedii* [18]. Specifically, the contents of ICA and the total flavonoids are no less than 0.5% and 5.0% of the components, respectively [16]. The main isolation methods for ICA include water boiling extraction, ethanol reflux extraction [19–22], and an ultrasonic-assisted ethanol extraction method developed by Zhang et al. [21]. The optimum ultrasonic-assisted extraction conditions were determined by an orthogonal test as follows: 50% (*v*/*v*) ethanol solution, 30 ml/g liquid-solid ratio, ultrasonic duration for 30 minutes, 50°C extraction temperature, and extraction for 3 times. Compared with the traditional water boiling extraction method, this kind of method has higher efficiency. In addition, microwave, high pressure, and vacuum reflux extraction methods have also been tested [17, 21, 23, 24]. *Herba epimedii* is widely used for the treatment of osteoporosis in China [18, 25, 26], and results from clinical trials [27, 28] show similar antiosteoporotic effects of its flavonoid extracts, as well as those of *Epimedium* total flavone capsules. The effects of ICA have largely been studied in animal or *in vitro* models [2, 4, 5, 9, 29, 30], and its potential clinical applications are rarely reported.

## 3. Role of BMSCs in Bone Regenerative Therapy

The common culture method of mouse BMSCs was as described in several studies [31–34]. Bone marrow is extracted from the femur and tibia of mice using an aseptic technique. The bone marrow is then cultured *in vitro* and subcultured to the third passage. As for human BMSCs, the proximal femur or posterior iliac crest is the common part from which to extract human bone marrow [30, 32, 33, 35]. The bone marrow is cultured *in vitro* and usually cultured to the third passage [33]. It should be noted that the cell phenotype identification is important in BMSC culture processes [32]. The sorted mouse CD29^+^Sca-1^+^CD45^−^CD11b^−^ BMSCs and human CD146^+^STRO-1^+^CD45^−^ BMSCs are cultured for 1-2 weeks to reach 80%-85% confluence [32, 33]. Then, first-passage BMSCs are detached and seeded in culture flasks for enrichment of cell populations.

The bone is a kind of mineralized connective tissue which exhibits four types of cells: osteoclasts, osteoblasts, osteocytes, and bone lining cells. Osteoblasts, which comprise 4-6% of the total bone cells, are located along the bone surface and are widely known for their role in bone formation [36]. The osteocytes accounting for 90-95% of the total bone cells are located within lacunae surrounded by a mineralized bone matrix wherein they exhibit a dendritic morphology [37]. The morphology of embedded osteocytes varies independently of bone types. Osteoclasts derive from mononuclear cells of the hematopoietic stem lineage which are multinucleated and terminally differentiated. Though it exhibits an inert appearance, bone tissue is constantly resorbed by osteoclasts and reformed by osteoblasts in a highly dynamic way. The process of bone remodeling is greatly complicated which is in a cycle comprising three stages: (1) initiation of bone resorption by osteoclasts, (2) transition between resorption and reformation, and (3) formation of new bone by osteoblasts. This bone remodeling process requires coordinated actions of osteocytes, osteoclasts, osteoblasts, and bone lining cells which together form the temporary anatomical structure called the basic multicellular unit [38, 39]. Osteoblasts are the main functional cells of bone formation, which are mainly responsible for the synthesis, secretion, and mineralization of the bone matrix. Osteoblasts can produce extracellular matrix proteins and mineralization regulators [38], during which period it undergoes significant proliferation and differentiation. Osteoclasts are the only cells with bone resorption function. Many cytokines such as interleukin-6 (IL-6), tumor necrosis factor-*α* (TNF-*α*), and cathepsin K can provide signals for osteoclast differentiation and bone resorption, promote the recruitment of osteoclast precursors, and drive osteoclast differentiation and bone resorption [40–43]. The osteoblasts and the differentiation of osteoclasts are regulated by many signaling pathways, among which the bone morphogenetic protein-drosophila mothers against decapentaplegic protein (BMP-Smad) signaling is important [44]. The bone mass can be increased by promoting the directional differentiation of BMSCs into osteoblasts, which is driven by the runt-related transcription factor 2 (Runx2) and Osterix (Osx) [36, 38, 45, 46]. Since osteocytes are derived from the BMSC lineage through osteoblast differentiation [38], so finding potential drugs prompting the differentiation of BMSCs into osteoblasts may be a promising strategy for bone regeneration.

Hematopoietic stem cells (HSCs) and BMSCs are the two pluripotent cell types found in the bone matrix [47, 48]. BMSCs were first isolated from the adult bone marrow [49] and can differentiate into the adipocytes, chondrocytes, osteoblasts, and myoblasts [29, 50, 51]. Therefore, BMSCs are a highly promising therapeutic option for cardiovascular, orthopedic, and joint degenerative diseases [52–57]. Several studies have examined the ability of BMSCs to improve bone formation and prevent bone loss and necrosis, in addition to ameliorating congenital bone defects and osteoporosis [58, 59]. However, the engrafted BMSCs have poor survival and a low rate of differentiation at the site of transplantation, which significantly reduce the efficacy of BMSC-based regenerative therapy. Therefore, it is essential to develop new drugs to enhance BMSC proliferation and differentiation.

**Figure 2:**
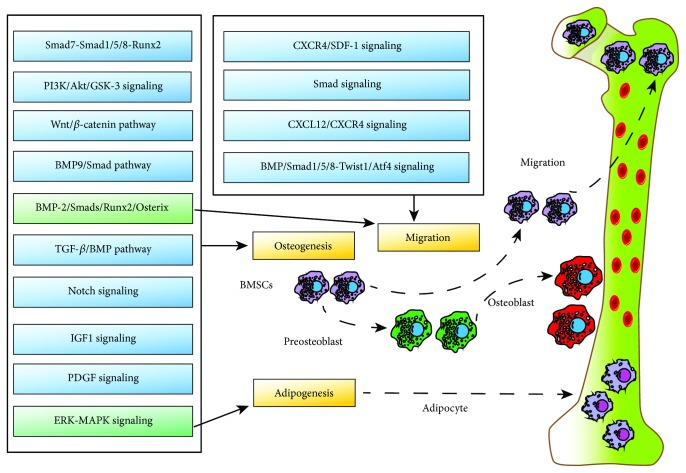
Signaling pathways involved in osteogenesis, adipogenesis, and migration regulation of BMSCs.

BMSC osteogenesis is the key step in bone regeneration and is affected by several factors including hormones, growth factors, environmental factors, and microRNAs [11]. BMSCs not only give rise to bone tissues but can also differentiate into adipose cells or osteoblasts [60]. Under physiological conditions, a dynamic balance exists between the osteogenic and the adipogenic potential of BMSCs [61–63] and is primarily regulated by Runx2 and the peroxisomal proliferator-activated receptor gamma (PPAR*γ*) [30, 64]. Runx2 is regulated by BMP-2 and is a key modulator of osteogenic differentiation, whereas PPAR*γ* promotes adipogenesis and inhibits osteogenesis [65–67]. Both signaling pathways concurrently regulate different cytokines to determine the fate of BMSC differentiation [68, 69]. The extracellular signal-regulated kinase-mitogen-activated protein kinase (ERK-MAPK) signaling pathway is also a key player in regulating BMSC differentiation [70], whereas the platelet-derived growth factor (PDGF) pathway is an essential proosteogenic pathway [71]. Cao et al. showed that Notch and BMP-9/Smad signaling synergistically enhanced osteogenic differentiation [72], and Li et al. found that miR-21 directly acted on Smad7 in the Smad7-Smad1/5/8-Runx2 pathway to modulate osteogenic differentiation [73]. Long et al. demonstrated that miR-139-5p regulated osteogenic differentiation of BMSCs via the Wnt/*β*-catenin pathway [74]. Furthermore, the transforming growth factor-*β*/bone morphogenetic protein (TGF-*β*/BMP) [73], phosphatidylinositol 3-kinase/protein kinase B/glycogen synthase kinase-3 (PI3K/Akt/GSK-3) [9], extracellular regulated kinase (ERK), PI3K/Akt [75], and insulin-like growth factor 1 (IGF1) [76] pathways also play important roles in osteogenic differentiation and bone formation (Figure 2). Since the two differentiation pathways are competing [68, 69], interregulatory, and interconvertible, certain growth factors can be used to promote the osteogenic differentiation of BMSCs ex vivo for bone tissue engineering.

Migration of BMSCs to the site of bone defect is a critical step in the treatment of orthopedic disorders [77]. Previous studies [78–81] have shown that the C-X-C motif chemokine ligand 12/C-X-C chemokine receptor type 4 (CXCL12/CXCR4) axis modulates BMSC homing and promotes angiogenesis, and the BMP-2/Smads/Runx2/Osterix axis modulates BMSC osteoblastic differentiation. The crosstalk between these two signaling axes is mediated by CXCR4, which modulates the migration [82] and osteogenic differentiation of BMSCs. Some studies have demonstrated that BMSC migration can also occur via the BMP-Smad1/5/8-twist-related protein 1/activating transcription factor 4 (Twist1/Atf4) [83, 84] and CXCR4/stromal-derived factor 1 (SDF-1) [85–87] axes and the Smad pathway [88].

## 4. Mechanisms of BMSC Regulation by ICA

### 4.1. BMSC Proliferation and Osteogenesis Promoted by ICA

ICA has multiple pharmacological activities, including hormone-like, antitumor, immunomodulatory, and antioxidative effects [89–94]. Studies [95, 96] show that ICA-mediated osteogenesis is associated with its hormone-like function. It can induce BMP-2 and BMP-4 mRNA expression in osteoblasts and significantly upregulates Osx at low doses [97, 98]. In addition, ICA facilitates bone formation by inducing proosteoblastic genes like Osx, Runx2, alkaline phosphatase (ALP), and collagen type I. It also inhibits bone resorption by regulating the osteoprotegerin/receptor activator of nuclear factor-*κ*b ligand (OPG/RANKL) signaling in the osteoclasts [99]. Zhang et al. found that ICA inhibits the adipogenic differentiation of BMSCs and promotes osteoblastic differentiation [100]. Fan et al. found that ICA promoted not only BMSC proliferation *in vitro* in a dose-dependent manner but also osteoblastic differentiation at very low doses (10^−9^ M to 10^−6^ M). However, a higher concentration of 10^−5^ M was toxic and suppressed osteoblastic differentiation [30]. Using a rat model of bone fracture, Cao et al. [2] showed that intragastric administration of ICA significantly increased osteotylus formation and accelerated bone healing within 5 months of treatment. These findings demonstrate that ICA administration following bone fracture can accelerate mineralization and osteogenesis and significantly improve bone healing. Therefore, ICA can also be an alternative treatment for postmenopausal osteoporosis and bone fracture.

The imbalance between BMSC adipogenesis and osteogenesis is considered the primary cause of femoral head necrosis [101]. The two processes are normally at an equilibrium under physiological conditions, which can be disrupted by external factors such as steroids and alcohol. Huang et al. [4] showed that ICA can effectively prevent femoral head necrosis, improve prednisolone-induced BMSC proliferation, enhance osteoblastic differentiation, and inhibit adipogenic differentiation. In addition, low concentrations of ICA (10^−9^ M to 10^−5^ M) significantly increased BMSC proliferation, especially at 10^−6^ M [4, 30].

Sun et al. [29] found that ICA restored the balance between osteogenic and adipogenic differentiation of mesenchymal stem cells in patients with osteonecrosis of the femoral head via ATP-binding cassette subfamily B member 1- (ABCB1-) promoter demethylation. In addition, ICA inhibited the differentiation of mesenchymal stem cells into adipocytes by inhibiting PPAR*γ*, CCAAT/enhancer binding protein *α* (C/EBP*α*), and fatty acid-binding protein 4 (FABP4) mRNA via the Notch signaling pathway [102]. Zheng et al. [103] also found that daily oral administration of ICA (0.3 mg/g) to osteoprotegerin knockout male mice for 8 weeks increased the trabecular bone volume and trabecular number, indicating an important role of osteoprotegerin in ICA-mediated osteogenic effects. In addition, osteocalcin and osteopontin also mediate ICA-induced osteogenic differentiation by increasing ALP activity and collagen type I levels [104]. These results [29, 102–104] indicated that ICA plays an important role in bone synthesis and metabolism. Furthermore, ICA significantly promoted bone healing by increasing BMSC proliferation and osteoblastic differentiation in a New Zealand rabbit model of bone defect [105]. ICA can also induce BMSC osteoblastic differentiation under various pathological conditions such as osteoporosis [5] and bone necrosis [29]. Estrogen and epigenetic regulation are currently the research focus of ICA-induced osteogenesis under pathological conditions [5, 29]. Sun et al. showed that ICA improved BMSC viability and osteoblastic differentiation by upregulating ABCB1, indicating a demethylating function as well [29]. In addition to promoting osteogenic differentiation of BMSCs, ICA can also promote bone regeneration by promoting angiogenesis [106], since vascularization is a key step in bone regeneration which recruits the BMSCs and essential factors to the site of trauma [107].

Icaritin is a biologically active metabolite of ICA [108] and can be easily extracted from various sources. Wu et al. [12] showed positive effects of icaritin on BMSC osteoblastic differentiation *in vitro*. It promotes osteogenic differentiation and inhibits adipogenic differentiation of BMSCs by inactivating GSK-3*β* and suppressing PPAR*γ* expression [102, 109, 110]. In addition, the BMPs (BMP-2, BMP-4, and BMP-7) and the MAPK/ERK pathway are also involved in icaritin-mediated osteogenic differentiation [12, 110].

Taken together, ICA promotes BMSC proliferation and osteoblastic differentiation and inhibits adipogenic differentiation, indicating its potential as a bone regenerative drug.

### 4.2. Signaling Pathways Involved in ICA-Mediated BMSC Proliferation and Osteogenesis

The MAPK pathway consists of the ERK, p38 kinase (p38), and Jun amino-terminal kinases/stress-activated protein kinase (JNK) factors. It regulates essential cellular functions, such as growth, proliferation, differentiation, and apoptosis. In addition, MAPKs also mediate the biological functions of ICA [111], indicating a possible role in BMSC proliferation as well. Qin et al. [112] found that ICA-induced rat BMSC proliferation *in vitro* was positively correlated with ERK levels and p38 phosphorylation and significantly upregulated Elk1 and c-Myc, the transcription factors downstream of the MAPK pathway.

A study using BMSCs extracted from SD rat bone marrow showed that 0.05-2.0 mg/l ICA significantly facilitated BMSC proliferation by activating the Wnt/*β*-catenin pathway [113]. Ye et al. [114] found that low doses of ICA (10^−8^ M to 10^−6^ M) promoted the proliferation and osteoblastic differentiation of rat adipose-derived stem cells (ADSCs), and 10^−7^ M ICA significantly upregulated RhoA (ras homolog gene family, member A) and p-MYPT1 (a ROCK or Rho-associated protein kinase substrate). This indicates that ICA promotes rat ADSC proliferation and osteoblastic differentiation by activating the RhoA-transcriptional coactivator with the PDZ-binding motif (TAZ) signaling pathway. Furthermore, Zhai et al. [115] showed the involvement of the PI3K/Akt/eNOS/NO/cGMP/PKG signaling pathway in the ICA-mediated osteogenesis of BMSCs. As already mentioned, any imbalance between BMSC osteoblastic and adipogenic differentiation impairs bone stability and leads to bone loss and increased bone marrow adipogenesis [116], resulting in osteoporosis and bone necrosis [117, 118]. TAZ is a *β*-catenin-like transcriptional coactivator involved in modulating this balance [119, 120]. It activates Runx2-mediated transcription to regulate BMSC differentiation and stimulate osteoblastic differentiation and also interacts with PPAR*γ* to suppress adipogenic differentiation. Furthermore, Wei et al. [121] demonstrated that ICA promotes BMSC proliferation and osteogenesis via activation of the estrogen receptor (ER)*α*-Wnt/*β*-catenin signaling pathway. There is considerable ambiguity regarding the interaction between TAZ and Wnt/*β*-catenin. One study [122] indicated an upstream regulatory role of TAZ, while another study [123] showed that TAZ lies downstream of the Wnt/*β*-catenin cascade. Nevertheless, TAZ is an important regulator of ICA-mediated BMSC osteoblastic differentiation.

**Figure 3:**
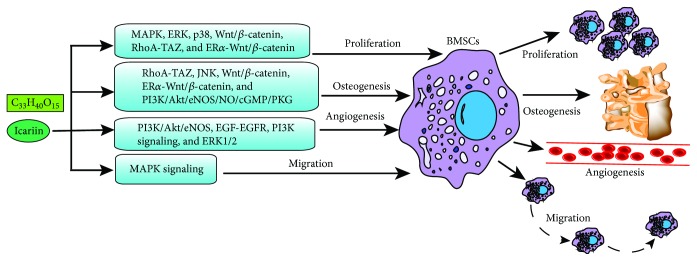
Signaling pathways involved in ICA-mediated BMSC proliferation, osteogenesis, angiogenesis, and migration. ICA promotes proliferation of BMSCs through signaling pathways such as MAPK, ERK, p38, Wnt/*β*-catenin, RhoA-TAZ, and ER*α*-Wnt/*β*-catenin. ICA promotes BMSC osteogenesis via signaling pathways such as RhoA-TAZ, JNK, Wnt/*β*-catenin, ER*α*-Wnt/*β*-catenin, and PI3K/Akt/eNOS/NO/cGMP/PKG. ICA promotes BMSC angiogenesis via PI3K/Akt/eNOS, EGF-EGFR, PI3K, and ERK1/2 signaling pathways. ICA promotes migration of BMSCs through the MAPK signaling pathway.

Kammerer et al. [124] reported that the ER*α* signaling pathway transcriptionally regulates Runx2, while Cai et al. [125] showed that the Wnt/*β*-catenin pathway directly targeted Runx2 to promote osteoblastic differentiation and the calcification of vascular smooth muscle cells. Both studies indicated a close association of the ER*α* and Wnt/*β*-catenin signaling pathways with the Runx2 expression. Another study [126] found that ICA stimulated BMSC osteoblastic differentiation by upregulating TAZ and the downstream osteogenic genes, and blocking the aforementioned pathways abrogated ICA-induced TAZ upregulation (Figure 3). These findings point to a TAZ/ER*α*/Wnt/*β*-catenin axis that mediates ICA-induced BMSC osteoblastic differentiation. In one study, Wu et al. [104] demonstrated the involvement of the JNK pathway in the osteoblastic differentiation of BMSCs or periodontal ligament stem cells [127, 128].

Multiple signaling pathways, including the BMP, nitric oxide (NO), MAPK, and Wnt/*β*-catenin pathways, are likely activated in the osteoblasts due to the estrogen-like properties of ICA and ICA-induced estrogen production [90, 129, 130]. Shi et al. [131] showed that ICA promoted osteogenesis in rat cranial osteoblasts and in an *in vivo* rat model of bone growth by activating the cAMP/PKA/CREB signaling pathway.

### 4.3. ICA Promotes BMSC Migration and Angiogenesis

ICA not only activates endothelial angiogenesis *in vitro* but also directly stimulates angiogenesis *in vivo*, through the PI3K/Akt/eNOS-dependent signaling pathways [106]. ICA can activate the epidermal growth factor-epidermal growth factor receptor (EGF-EGFR) pathway to promote endothelial NOS synthesis, thereby facilitating vascular regeneration [132]. In addition, ICA can directly stimulate angiogenesis by activating various angiogenic factors like ERK, PI3K, and Akt [133, 134]. An *in vitro* study by Liu et al. [135] showed that ICA upregulated angiogenesis-related genes like vascular endothelial growth factor (VEGF) and fibroblast growth factors (FGF). In addition, ICA upregulated brain-derived neurotrophic factor (BDNF) and VEGF via the PI3K and ERK1/2 signaling pathways [134], which in turn promoted the angiogenic differentiation of BMSCs. Furthermore, Jiao et al. [136] found that ICA enhanced the migratory ability of BMSCs *in vitro* and *in vivo*, via the MAPK signaling pathway.

In summary, the primary effects of ICA in BMSCs are to promote proliferation and osteogenesis and are mediated by multiple signaling pathways including the MAPK/ERK/p38, Wnt/*β*-catenin, PI3K/Akt/eNOS/NO/cGMP/PKG, RhoA-TAZ, and ER*α*-Wnt/*β*-catenin pathways. In addition, ICA can also act on osteoblasts through the BMP/Runx2, NO, MAPK, Wnt/*β*-catenin, cAMP/PKA/CREB, and JNK pathways. Furthermore, ICA promotes angiogenesis via the PI3K, ERK1/2, and EGF-EGFR pathways and BMSC migration via the MAPK pathway. The angiogenic effect of ICA is favorable for osteogenesis, although their exact relationship as well as that between angiogenesis and migration still needs to be elucidated.

## 5. Prospects

ICA can significantly promote BMSC proliferation and osteoblastic differentiation and inhibit adipogenic differentiation, making it a reliable option for bone regenerative therapy. Mechanistic studies show that multiple signaling pathways mediate these processes, indicating the potential of multiple therapeutic targets. Above all, ICA could be made into a liquid state at suitable concentration in the future and be applied in bone regeneration. Besides, the evidence indicated that the optimal concentration for ICA which can perform better effects in BMSCs is 1 *μ*M [4, 30], while others reported 0.1 *μ*M [114, 121, 126]. However, further studies are needed to figure out both a safe and an effective concentration of ICA [14]. For patients with fractures, bone defects, nonunion disorders, and osteonecrosis of the femoral head, a mixture of ICA and autologous BMSCs can be injected locally into the lesion to facilitate bone regeneration. For patients with osteoporosis, ICA can be delivered through intravenous administration. Since ALP (an early marker of osteogenic differentiation) levels peak on the 14^th^ day of the *in vitro* BMSC culture with ICA [121, 126, 135], it is reasonable to consider 14 days of intravenous ICA administration for the treatment regimen. ICA also promotes the regeneration of periodontal tissue [137], peripheral nerves [138], neural stem cells [139], and endometrium [140], although the optimal concentration of ICA differs across tissues.

There are still several questions that need to be addressed in future studies. For example, although the pathways involved in BMSC migration are well-known, the mechanism(s) underlying ICA-mediated BMSC migration remain to be elucidated. At present, it is not clear whether there is a synergistic or antagonistic crosstalk or upstream and downstream relationship among the signaling pathways involved in ICA-mediated osteogenic differentiation of BMSCs. Most studies on BMSC osteogenesis and migration have been carried out under normoxic conditions. The oxygen levels in ischemic lesions, such as in femoral head necrosis, can be less than 1% [141], and severe hypoxia affects the osteogenic differentiation and migration of BMSCs *in vivo* and *in vitro* [142, 143]. Therefore, it is necessary to simulate the hypoxic conditions in the *in vitro* studies.

In conclusion, a better understanding of the role and mechanism of action of ICA in BMSCs can provide new therapeutic strategies for various orthopedic disorders such as osteoporosis, femoral head necrosis, and bone defects.

## Abbreviations

BMSCs: Bone marrow mesenchymal stem cells
ICA: Icariin
IL-6: Interleukin-6
TNF-1: Tumor necrosis factor
PPAR*γ*: Peroxisomal proliferator-activated receptor gamma
Runx2: Runt-related transcription factor 2
BMP-2: Bone morphogenetic protein-2
ERK-MAPK: Extracellular signal-regulated kinase-mitogen-activated protein kinase
ALP: Alkaline phosphatase
OPG/RANKL: Osteoprotegerin/receptor activator of nuclear factor-*κ*b ligand
C/EBP*α*: CCAAT/enhancer-binding protein *α*
FABP4: Fatty acid-binding protein 4
PDGF: Platelet-derived growth factor
CXCL12: C-X-C motif chemokine ligand 12
CXCR4: C-X-C chemokine receptor type 4
SDF-1: Stromal-derived factor 1
Smad: Drosophila mothers against decapentaplegic protein
Twist1: Twist-related protein 1, also known as class A basic helix-loop-helix protein 38 (bHLHa38)
Atf4: Activating transcription factor 4
TGF-*β*/BMP: Transforming growth factor-*β*/bone morphogenetic protein
PI3K/Akt/GSK-3: Phosphatidylinositol 3-kinase/protein kinase B/glycogen synthase kinase-3
ERK: Extracellular signal-regulated kinase
IGF1: Insulin-like growth factor 1
Osx: Osterix
EGF-EGFR: Epidermal growth factor-epidermal growth factor receptor
NO: Nitric oxide
VEGF: Vascular endothelial growth factor
FGF: Fibroblast growth factors
MAPK: Mitogen-activated protein kinase
p38: p38 kinase
JNK: Jun amino-terminal kinases/stress-activated protein kinase
RhoA: Ras homolog gene family, member A
p-MYPT1: A ROCK (Rho-associated protein kinase) substrate molecule
ER*α*: Estrogen receptor *α*
PI3K/Akt/eNOS/NO/cGMP/PKG: Phosphatidylinositol 3-kinase/protein kinase B/endothelial nitric oxide synthase/nitric oxide/cyclic guanosine monophosphate/protein kinase-G
cAMP/PKA/CREB: cAMP/protein kinase A/cAMP response element-binding protein
GSK-3: Glycogen synthase kinase-3
ABCB1: ATP-binding cassette subfamily B member 1
TAZ: Transcriptional coactivator with PDZ-binding motif
BDNF: Brain-derived neurotrophic factor.
